# Magnetoencephalography and High-Density Electroencephalography Study of Acoustic Event Related Potentials in Early Stage of Multiple Sclerosis: A Pilot Study on Cognitive Impairment and Fatigue

**DOI:** 10.3390/brainsci11040481

**Published:** 2021-04-09

**Authors:** Damiano Paolicelli, Alessia Manni, Antonio Iaffaldano, Giusy Tancredi, Katia Ricci, Eleonora Gentile, Rosa Gemma Viterbo, Silvia Giovanna Quitadamo, Marina de Tommaso, Maria Trojano

**Affiliations:** 1 Department of Basic Medical Sciences, Neurosciences, and Sense Organs, University of Bari “Aldo Moro”, 70121 Bari, Italy; ali.manni@libero.it (A.M.); antonioiaffaldano@yahoo.it (A.I.); tancredi.giusy.96@gmail.com (G.T.); katiari86@gmail.com (K.R.); rosa.viterbo@uniba.it (R.G.V.); sg.quitadamo@gmail.com (S.G.Q.); marina.detommaso@uniba.it (M.d.T.); maria.trojano@uniba.it (M.T.); 2 Basic Health District, Family Counseling Center, ASP (Local Health Company), 85038 Senise, Italy; eleonora.gentile.psy@gmail.com

**Keywords:** multiple sclerosis, early stage, MEG, EEG, event related potentials, fatigue, cognitive impairment

## Abstract

Cognitive impairment (CI) is a common and disabling symptom of Multiple Sclerosis (MS) with a negative impact on daily living. In this pilot study, we applied magnetoencephalography (MEG) and high density (hd) electroencephalography (EEG) study to evaluate acoustic P300 features in a cohort of early MS. Sixteen MS patients (pwMS) and 19 healthy controls (HCs) matched for age and gender underwent an MEG-/(hd)-EEG-co-recording, using 306-channel Vectorview and 64 scalp electrodes. CI was assessed using Rao’s Brief Repeatable Battery (BRB). Moreover, we performed psychometric tests to assess depression and fatigue. In pwMS, we observed a slight latency prolongation of P300 peak compared to HCs, while P300 amplitude and scalp distribution were similar in the two groups. pwMS did not show an amplitude reduction and different scalp distribution of Event-Related Potentials (ERPs) and Event Related Fields (ERFs) related to an acoustic oddball paradigm. We found an inverse correlation between P300 amplitude and fatigue (r Spearman = −0.4; *p* = 0.019). In pwMS, phenomena of cortical adaptation to early dysfunction could preserve the cognitive performance of the P300 acoustic task, while the development of fatigue could prospectively lead to amplitude decline of P300, suggesting its possible role as a biomarker.

## 1. Introduction

Cognitive impairment (CI) is a common and disabling symptom of Multiple Sclerosis (MS). Its prevalence ranges from 43% to 70% of MS patients (pwMS) [1]. Recent data suggest that, although CI has been reported in all disease phenotypes, disease duration and age play an important role in the cognitive profile of pwMS [2]. In fact, the prevalence of CI is higher at later stages of relapsing-remitting (RR) MS and in progressive forms of the disease [2,3], but cognitive dysfunctions were also detected both in patients with Clinically Isolated Syndrome (CIS) and in the early stages of relapsing forms [4,5]. CI in MS involves multiple cognitive domains: Episodic memory and information processing speed are mostly affected, but deficits in working memory, executive functions, verbal fluency, and attention may also be present [1,6]. Some evidences suggested that early CI is a bad prognostic factor being associated with higher risk of conversion to definite MS [7], disability progression, transition to progressive phase, and cortical thinning [8]. CI has been recognized in patients with pediatric multiple sclerosis in more than 30% of cases [9].

Furthermore, CI can have a negative impact on daily life, including social relationships, employment, and overall quality of life, regardless of patients’ physical disability [10,11]. For these reasons, it is desirable to recognize CI as early as possible to personalize therapeutic strategies. Therefore, neuropsychological testing batteries tailored to target the key areas of MS cognitive dysfunction, such as the Brief Repeatable Battery of Neuropsychological Tests (BRB-N), are typically used in research settings [6] and in clinical practice too, although their use can be limited by the long administration time, practice effect, and physical disability [12].

Another common symptom, with an estimated prevalence of up to 83%, is fatigue, which exerts the greatest impact on patients’ quality of life [13]. Many mechanisms may be related to fatigue in MS: structural damage of white matter (WM) and grey matter (GM) [14,15], brain atrophy [16], functional brain connectivity changes [17], disease worsening [18], compensatory brain over activation patterns [19], depression [20]. In previous works, the damage of different brain structures has been related to fatigue and depression in MS. Moreover, fatigue was associated with clinical disability [21] and also with cognitive performance. During a cognitive task, the impact of fatigue, measured by the Modified Fatigue Impact Scale (MFIS), on cortical activation, is demonstrated by the incongruity of the activated areas depending on the MFIS score [22]. The most applied tools used to assess fatigue in MS clinical settings, thanks to its high specificity and sensitivity in distinguishing pwMS from healthy controls (HCs), are the fatigue severity scale inventory (FSS) [23] and the fatigue scale for motor and cognitive functions (FSMC) [24]. Despite its frequency and pronounced impact on the lives of pwMS, techniques for differential diagnosis of fatigue and mechanism-guided treatment selection in individual patients do not exist [13]. The identification of measures of CI and fatigue able to overcome these limitations is an open challenge in the routine clinical setting.

Functional Magnetic Resonance Imaging (fMRI), a technique based on metabolic changes, was the main technique for the study of the functional network organization in MS. Magnetoencephalography (MEG) represents another method to quantify functional networks, which, unlike the fMRI, directly measures neural activity. MEG has an excellent temporal resolution, and recently, its spatial resolution has greatly improved [25]. MEG, compared to fMRI, according to a recent study, has a higher sensitivity in detecting relevant cognitive disruptions in functional networks [26].

The fMRI also failed to identify alterations in the neuronal organization during early MS, which, instead, was done by electrophysiological investigations [26]. Investigations of electrophysiological, spectrally-resolved functional connectivity (FC) with MEG or electroencephalography (EEG) have been, for these reasons, progressively investigated.

In addition to neuropsychological tests, P300 event-related brain potentials (ERPs) have been used as neurophysiological markers in the assessment of cognition in patients with relapsing-remitting multiple sclerosis (RRMS) research [27,28] and, more recently, also in clinical practice [29]. Their measurement can clarify cognitive processing phases as encoding, selecting, memorizing, and decision making. Their magnetic counterpart (Event Related Fields—ERF) provides advanced topographic analysis and further data on subtle modifications of cognitive induced changes of brain activity. Due to the ease of recording and reliability, the P300 has become the most studied endogenous ERPs component. Results of many ERPs studies are consistent with prolonged P300 latencies [30,31,32,33,34,35,36,37] and/or smaller P300 amplitudes [30,33,36] in 50–65% of pwMS [38]. Significant correlations are reported between prolonged P300 latency and poorer scores on neuropsychological tests, as BRB, Mini Mental State Examination (MMSE), Wechsler adult intelligence scale (WAIS), and others [30,32,34,37], Expanded Disability Status Scale (EDSS) scores [31,35,36,37], disease duration [31] and Magnetic Resonance Imaging (MRI) lesion load [32,37]. Although the P300 latency and amplitude are progressively impaired in the course of MS, especially in the secondary progressive phenotype [38], their abnormalities were also observed in early RRMS [39,40] and CIS patients [41,42]. In addition, event-related spectral perturbations (ERSPs; event-related mean power spectral changes) could reveal subtle changes of EEG activity under P3b protocol, as demonstrated in the advanced phase of MS [43].

Only the acoustic ERPs are used in the study and not the visual ones. The choice was made to eliminate the influence of visual deficits, assuming greater visual damage in the course of MS, which have a greater impact on ERP anomalies [44,45].

To reveal early cortical dysfunction in the initial and non-medicated phase of MS, this pilot study focused on:

- Advanced topographic analysis of ERP and ERF and related ERSPs related to an acoustic oddball paradigm in a cohort of early and treatment-naïve pwMS;
- Correlations between P300 features with demographic, clinical, and neuropsychological characteristics, including fatigue, depression, and cognitive impairment.

## 2. Materials and Methods

### 2.1. Study Design and Population

This is a clinical, cross-sectional, pilot study, conducted according to the International Conference on Harmonization Guidelines for Good Clinical Practice and the Declaration of Helsinki (World Medical Association Declaration of Helsinki (2017) Ethical principles for medical research involving human subjects). The study protocol was approved by the local Ethics Committee. All patients provided written consent. Demographic and baseline clinical data were collected at enrolment. The experimental session started at 8:30 AM and included:

- A complete neurological examination with disability assessment by EDSS [46]
- A 45-min neuropsychological evaluation
- A simultaneous high density (hd) MEG and EEG recordings (50–55 min preceded by 20–25 min setup time) of event related fields/potentials (ERF/ERP) and resting state data.

The present study focused on the auditory oddball task, chosen because the auditory system is less involved in all the phases of MS, enabling the extraction of late cortical potentials with latency and amplitude not affected by the dysfunction of the afferent pathways [47].

The study sample consisted of pwMS (*n* = 17) and HCs (*n* = 19) matched for age, sex, and education, enrolled at the Centre for Diagnosis and Treatment of Demyelinating Diseases, University Hospital of Bari, between March 2018 and April 2019. The minimum enrollment age was 18 years, while the upper age range limit was set to 55 years to avoid potential aging-related confounding factors, such as leukoaraiosis [48]. PwMS were selected across not active clinical phenotypes according to Lublin’s new MS phenotypic classification [49], with a diagnosis of CIS or RR disease, according to the revised McDonald criteria [50]. All subjects were minimally impaired and with a recent diagnosis. A disease history >5 years and a EDSS score >6 represented exclusion criteria. All participants were disease modifying drugs (DMDs) naïve. Moreover, pwMS were excluded from the study if they experienced a relapse or underwent steroid treatment 30 days before the examination. Relapses were defined as episodes of neurological symptoms occurring at least 30 days after the onset of any previous episode, lasting at least 24 h, not attributable to any other causes, occurring in the absence of an infection or fever, and accompanied either by new clinical signs, i.e., changes in the neurological examination, or by an increased EDSS score [51]. Exclusion criteria for all participants were history or presence of psychiatric or neurological disease (apart from MS for the patient group), as well as an antidepressant and antipsychotic therapies, drug and alcohol abuse, major traumatic head injury, moderate to severe hearing loss, vascular and metabolic conditions (ischemic heart disease, high blood pressure, type 1 and 2 diabetes, dyslipidemia, obstructive sleep apnea). Moreover, individuals with head and neck tattoos and metal implants were excluded due to safety issues.

Due to technical issues, the final population consisted of 16 pwMS and 18 HCs.

### 2.2. Neuropsychological Evaluation

All participants were administered Rao’s BRB-N [52] to evaluate verbal memory (Selective Reminding Test—Long-Term Storage, SRT-LTS; SRT—Consistent Long-Term Retrieval, SRT-CLTR; and SRT—Delayed recall, SRT-D), visuospatial learning (10/36 Spatial Recall Test, SPART; Spatial Recall Test—Delayed recall, SPART-D), information processing speed and attention (Symbol Digit Modality Test, SDMT; 3 and 5 s Paced Auditory Serial Addition Tests, PASAT-3 and PASAT-5), and verbal fluency (Word List Generation, WLG), as well as the Trail Making Test (TMT), to assess visual processing speed and executive functions [53]. A test was considered to be altered when the z-scored test results fell below two standard deviations (SD) from the normative mean values for the Italian population [54]. The threshold for the diagnosis of cognitive impairment was set to two altered tests (BRB-N and/or TMT). A global cognitive impairment index (GCI) was additionally calculated by assigning an integer value to each z-scored test result, corresponding to the number of SD from the normative mean values it fell within and computing the sum of these values. In addition, we assessed psychometric tests for measuring the severity of depression (Beck Depression Inventory, BDI) [55] and the level of fatigue by the use of FSS [23], with a cut-off value of 9 and 4.5, respectively, to identify individuals with even mild depressive symptoms or fatigue impacting daily activities.

### 2.3. P300 Procedure

The standard auditory oddball paradigm was performed with a stimulus sequence consisting of 240 standard tones (250 Hz) and 60 target tones (1000 Hz) presented binaurally via headphones, in a pseudorandom order, at 75 decibels. Stimulus duration was 100 ms, with stimulus onset asynchrony was between 2 and 3 s. Participants were given a brief practice session to clarify the distinction between target and standard stimuli. They were instructed to press a button as soon as possible following the high-frequency target tone. Speed and accuracy of response were emphasized equally in the task instructions. Task duration was 12 min and 33 s, following 5 min of resting state.

### 2.4. Simultaneous MEG and EEG Recording

Measurements were carried out with the Elekta Neuromag^®^ TRIUX system (Elekta Neuromag Oy, Helsinki, Finland), a 306-channel Vectorview whole head MEG device that comprises 102 planar gradiometers, 102 axial gradiometers, and 102 magnetometers in a helmet-shaped array. The built-in 64-channel electrode cap, connected to an amplifier, was used to record EEG and MEG simultaneously. The EEG reference electrode was attached to the linked mastoids, tip the ground electrode was placed at the left wrist. The head position relative to the sensor array was determined by five head-position indicator coils positioned on the scalp surface, three of them close to the hairline in the frontal region and the other two over the left and right mastoid, respectively. Additionally, vertical electrooculogram (EOG) and electrocardiogram (ECG) derivations were recorded simultaneously as auxiliary channels. Data were recorded at a sampling rate of 1024 Hz in a dimly lit and electrically and magnetically shielded room (ETS-Lindgren Euroshield, Eura, Finland). The participants were comfortably seated with their eyes open. Stimulus onset and reaction times were acquired in addition to electrophysiological data during task performance.

### 2.5. Artifact Correction and Data Analysis

Event related potentials (ERP) and event related fields (ERF).

Elekta Neuromag MaxFilter 2.2 temporal Signal Space Separation (tSSS) was applied with standard parameters to perform noise reduction and head movement compensation and automatically detect and correct bad MEG channels. Data were low-pass filtered at 330 Hz and high-pass filtered at 0.1 Hz. Further preprocessing relied on Brainstorm12 in the MATLAB^®^ environment (R2016b, The MathWorks Inc., Natick, MA, USA). Recordings, downsampled to 500 Hz, were visually inspected to remove bad segments and EEG bad channels. Stereotypical artifacts of non-neural origin (from ocular, cardiac, and muscular sources) were removed by applying the logistic infomax algorithm implemented in the runica function for MATLAB^®^13. Continuous data were parsed into epochs of 1000 ms duration, extending from −200 to 800 ms relative to stimulus onset, baseline corrected to the 100 ms immediately preceding the sound onset, and averaged. The N1, P1, N2, P3, and N4 peaks latencies were automatically searched at the electrodes FCz and Pz after applying a 30 Hz low-pass Kaiser filter. Specifically, the five ERP components were identified as the local minima (for negative peaks) or local maxima (for positive peaks) in the 40–140, 70–210, 160–290, 220–420, and 350–700 ms windows after stimulus onset, respectively. Peak latencies were visually checked and corrected if needed. Peak-to-peak amplitudes were measured for each ERP component relative to the preceding peak (relative to baseline for N1). In the present study, we report results concerning P300 features.

The P300 wave, in this pilot study, was investigated on a single channel (Pz channel, latency, and N2/P3 peak to peak amplitude). Topographic differences in the amplitude of the P300 and its magnetic counterpart M300 were assessed on the whole set of sensors, averaging the signal across the 220–420 ms post-stimulus interval.

#### Time-Frequency (TF) Analysis

The purpose of this analysis was to study oscillations that are not strictly locked in phase across trials. Averaging trials in the time-frequency domain allow extracting the power of the oscillations at different frequencies regardless of the phase shifts.

The time-frequency (TF) decomposition was based on the convolution of the signal with series of complex Morlet wavelets, which are sinusoidal waves, weighted by a Gaussian kernel. A family of Morlet wavelets is represented by a mother wavelet and a series of scaled and shifted versions of the mother wavelet. We considered that more cycles correspond to higher frequency precision and worse temporal precision and fewer cycles to better temporal precision and worse frequency precision.

So far, to get a good balance between temporal and frequency precision, we selected a central frequency (the frequency of the mother wavelet) of 1 Hz and a time resolution (FWHM) of 2 s (the complex Morlet wavelet model is described in the Supplementary Figure S1). To avoid edge effects, we segmented the data in the [−1200 1800] ms window around each stimulus onset before TF decomposition and then discarded 1000 ms from each side to get the TF maps relative to the [−200 800] ms window. Moreover, we removed the evoked response from each trial to bring the signals to a slightly more stationary state before computing TF. We normalized each frequency separately with respect to the baseline, [−200–2] ms from stimulus onset, to obtain more readable TF maps. Given that the signal carries a lot less power in the faster oscillations than in the slow oscillations (1/f decrease in power), we would always see values close to zero in the higher frequency ranges when representing the TF maps with a linear color scale. Therefore, we normalized the TF values by evaluating the deviation from the mean over the baseline, in percent: (ҳ − mean)/mean × 100). This normalization method is called event-related spectral perturbation (ERSP). Finally, although we used a linear frequency definition of 1:1:60 Hz to compute the TF maps, to perform the cluster-based statistical non-parametric mapping, we had to group by frequency bands because of computational limitations of the virtual machine. Therefore, the frequency definition of the statistical maps was the following: Delta, 1–4 Hz; theta, 5–8 Hz; alpha, 9–12 Hz; beta1, 13–16 Hz; beta2, 17–30; gamma, 31–45 Hz. We did not include higher frequencies, which are considered not to be as reliable for scalp data.

### 2.6. Statistical Analysis

Between groups, differences in demographic variables, as well as differences in P300 amplitude and latency, computed on the Pz electrode, were investigated by means of Univariate ANOVA and z score included in the SPSS statistics, version 21. The effect of sex and age was taken into consideration introducing sex as factor analysis and age as a covariate in the ANOVA; their effect was not relevant in the ANOVA analysis both in the neurophysiological and neuropsychological domain.

For topographic analysis, cluster-wise correction for multiple comparisons was achieved separately for electrodes, gradiometers, and magnetometers, using the ft timelockstatistics.m field trip function in Brainstorm [56].

For TF analysis, cluster-based statistical non-parametric mapping was applied.

For all tests, the *p*-value threshold was set at 0.05.

Correlations between P300 amplitude and latency and age, SDMT-PASAT, BDI, and FSS scores were tested by means of multiple linear regression analysis, included in the SPSS software, version 21.

## 3. Results

Demographic and baseline clinical characteristics of the study participants are provided in Table 1. In our cohort, we observed a slightly but not significantly higher percentage of males among HCs (12.5% vs. 31.6%; *p* = 0.19). pwMS had an EDSS score between 0 and 3.0 with minimal neurological impairment and short disease history.

Considering the cognitive evaluation, six patients (37.5%) had an alteration in at least one and three (18.7%) in at least two cognitive tests. Cognitive tests WLG and PASAT 3 were the most affected in pwMS (18.7%), followed by PASAT5 and SPART-D in 12.5%, SDMT, and TMT-B 6.2% (Table 2).

In the control group, two subjects (13.3%) showed an alteration in at least one test and only one subject in at least two tests (6.7%).

Comparing the scores of the individual tests between the two groups, a statistically significant difference was observed for the TMT-A test alone, with a median score higher in the patient group than in the controls (median [interquartile range]: 37 [31.2–45] sec., 26 [24–35] sec., respectively, *p* = 0.03). The patient group had a higher median CII (9 [5.5–13.5]) than healthy subjects (3 [2–11.5]), but this difference did not reach statistical significance (*p* = 0.16).

Taking into consideration the four cognitive domains selected during the analysis, the domain of attention and speed of information processing was the most affected in the patient group, presenting altered in 18.7% of cases. In the control group, two subjects (13.3%) showed alterations in verbal fluency and one subject (6.7%) in the attention/processing speed domain. In addition, patients presented a median overall score in the attention domain and processing speed significantly lower than that of healthy subjects (121 [122–142], 157 [125.5–165] respectively, *p* < 0.01), with no statistically significant differences in the remaining domains (Table 3).

Globally, pwMS presented with lower scores in SDMT-PASAT tests compared to HCs. None of the patients showed mild, moderate, nor severe depression on BDI tests. However, BDI scores were significantly lower in HCs than in pwMS (2.72 ± 1.13 vs. 6.25 ± 1.21; *p* = 0.041); FSS scores were significantly lower in HCs than in pwMS too (1.14 ± 0.408 vs. 2.63 ± 0.433; *p* = 0.017).

We observed a slight and not significant *P300 latency* prolongation in pwMS compared to HCs.

**Figure 1:**
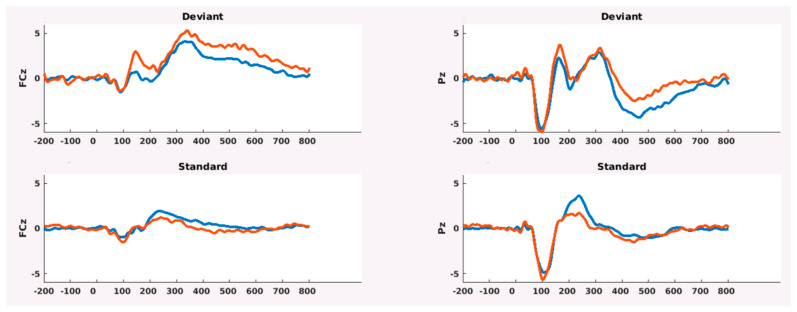
Grand Average of P300 in 19 HCs (blue trace) and 16 pwMs (red trace). Values are represented in uV.

The amplitude of P300 was similar between patients and controls (Table 4, Figure 1) as well as the topographical distribution of P300 and M300 patients displayed a slight increase in P300 representation over parietal electrodes and M300 over temporo-central electrodes.

**Figure 2:**
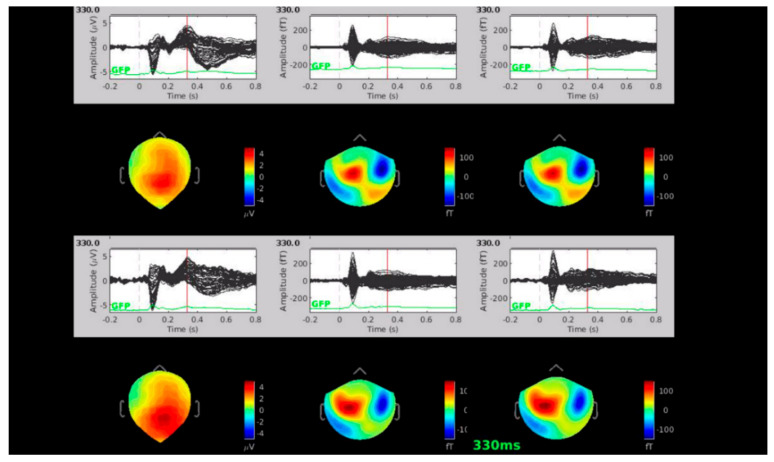
Topographical representation of Grand Average of P300 peak in 16 pwMS (above) and 19 HCs (below).

No relevant differences in topographical distribution emerged between pwMS and HCs (Figure 1 and Figure 2).

From left to right: EEG-ERP, MEG-magnetometers-ERF, MEG-gradiometers-ERF. No relevant differences of topographical distribution emerged between pwMS and HCs, apart from a slight prevalence of parietal P300 representation in pwMS.

The other considered peaks were not dissimilar from HCs (Supplementary Figures S2–S4). Time for motor responses (RT) was not significantly prolonged in pwMS (402.14 ± 30.31 msec vs. 375.5 ± 13.76; F: 0.1 *p* = 0.74). For five controls and two patients, a technical problem occurred in the codification of motor response.

**Figure 3:**
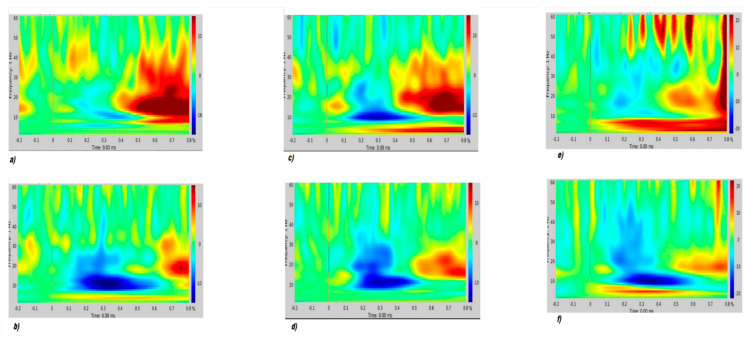
Standard trial. Representative parietal midline gradiometer (**a**,**b**), magnetometer (**c**,**d**) and electroencephalography (EEG) derivation (**e**,**f**) for HCs (top) and pwMS (bottom).

**Figure 4:**
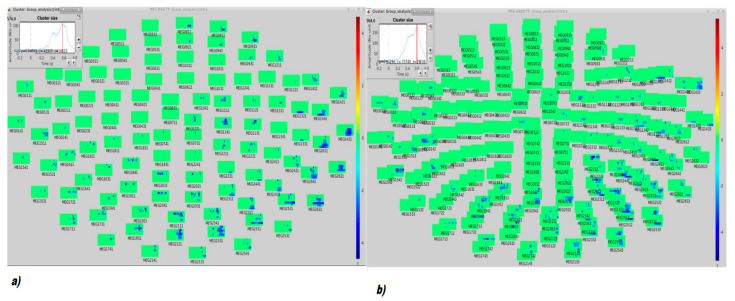
Topographical (and temporal, top left inset) distribution of the significant cluster (for (**a**) gradiometer, *p* = 0.049; (**b**) magnetometer *p* = 0.04).

Regarding TF analysis, for all sensor modalities, both control participants and patients showed in the standard condition a pattern of early negative modulation of the induced power in the alpha range (event-related desynchronization, ERD) and late positive modulation of the induced power in the beta range (event related synchronization, ERS). There was also a positive modulation of the induced power in the delta range (ERS), though the time specificity is more difficult to evaluate for these low frequencies. There was less positive and more negative variation in patients as compared to healthy controls. This difference reached significance for gradiometers and magnetometers while it was at a trend level for the EEG sensors (Figure 3 and Figure 4).

For the Deviant condition, both control participants and patients showed a pattern of early positive modulation of the induced power in the theta range (ERS), shortly followed by a prolonged negative modulation of the induced power in the beta range (ERD). Qualitatively, the positive variations with respect to the baseline were less positive, and the negative variations were more negative in patients as compared to healthy controls. However, there was no statistically significant difference. The Deviant–Standard interaction showed a pattern similar to the one seen for the Deviant condition with no significant differences between groups (Supplementary Figure S5).

We did not find a significant correlation among P300 features, demographic and neuropsychological variables in the groups of patients and controls, except for higher fatigue scores corresponding to reduced P300 amplitude (P300 amplitude vs. age, SMDT-PASAT, BDI, FSS multiple regression analysis in the total of patients and controls: corrected *r* square = 0.031 F = 1.2 *p* = 0.3; single coefficient FSS: *t* = −2.32 *p* = 0.048) (Table 2).

## 4. Discussion

Our main aim was to analyze P300 parameters, assessed through a multimodal MEG/ hd-EEG approach, and verify their possible correlation with CI and fatigue in pwMS. Moreover, for the first time to our knowledge, we investigated the early stages of the disease, considering DMDs naïve and minimally impaired subjects, with a short time to onset. Previous ERP studies were conducted in more advanced stages of MS when demyelination affects the afferent pathways, particularly the visual ones. The auditory system chosen in our work, instead, is less involved in all phases of MS, enabling the extraction of late cortical potentials with latency and amplitude not affected by the dysfunction of the afferent pathways. This may be due to the greater length of the sensory and motor pathways than the acoustic one in its course along the brain stem, but also, less likely, to a greater susceptibility of some pathways to the demyelinating process [47].

Our results show that main P300 parameters are within normal limits in patients with early MS, though they are affected by mild CI. We found a different scalp representation of spectral component perturbation following standard stimulus, with a similar trend for the deviant stimulus, between patients and controls.

Previous data from literature [30,33,36] have shown the prolonged latency of P300 in pwMS and its correlation with CI; only in one work, however [27], patients at the early stages of the disease, such as those diagnosed with CIS with the older McDonald’s criteria [57] were considered, among others, confirming P300 latency prolongation in the whole MS group. In our work, instead, the latency of P300 was prolonged but remained within normal limits.

Moreover, we found no differences in the amplitude and topographical distribution of N2-P3 complex and P3 peak between pwMS and HCs, different from existing literary data. In other works, in fact, ERP amplitude increases in patients with normal cognitive performance [58], and it is reduced in those with cognitive decline and the initial process of cortical atrophy [59]. An increase in P300 amplitude could imply, in later stages of the disease, mechanisms of cortical compensation and plasticity [58], which had probably not yet occurred in the selected MS populations, who were evaluated at a very short time from the disease onset, and had mild CI. On the other hand, our early MS patients did not show a P300 amplitude decrease because of the recent pathological process. Therefore, in our population, P300 amplitude could not be a pattern of early cortical dysfunction, though in the presence of mild CI. The hd EEG and MEG confirmed the normal scalp distribution of P300 waves, with slight differences in topographical representation, as we demonstrated by employing high resolution topographical analysis, which enabled the detection of different ERP/ERF scalp distribution. Most P300 hd studies relied on EEG methods [59,60]. However, the magnetic counterpart of the EEG P300 unveils the complexity of this potential. According to previous studies [61], EEG showed a centro-parietal representation of P300, which was similar in pwMS and HCs. The ERF was maximally represented on the central, parietal, and prefrontal regions, but no clear peaks were detectable; rather they were quite dispersed, suggesting a multisource origin of the magnetic fields [62]. The topographical analysis we employed was performed for every time unit in pwMS and HCs, and it did not show relevant differences between groups. Patients displayed a slight increase in P300 representation over parietal electrodes and M300 over temporo-central electrodes.

As a matter of fact, we can assume that in this small MS cohort, the ERP and ERF generated during an oddball paradigm have a similar scalp distribution in early pwMS and HCs. However, time-frequency analysis showed a different cortical activation in pwMS during standard stimulus processing, with a similar trend for the deviant stimulus. In TF analysis, MEG performed better than EEG in revealing subtle differences in early MS. These slight abnormalities confirm that in the early phase of demyelinating disease, latent rearrangement in cortical activation could preserve cognitive processes. The ERSPs on 128 EEG channels performed in more advanced MS patients showed reduced P3b task-related theta power, a pattern possibly emerging during the development of the disease [43].

The last objective of our study was to examine how depression, fatigue, and CI can affect P300 features. Our early MS patients displayed mild CI, according to previous studies. The oddball paradigm we performed was correctly executed by pwMS, as also demonstrated by the time of the motor response. However, there was a mild and not significant prolongation of reaction time, in line with the mild P300 latency increase. In this small early MS cohort, the cognitive performances subtending the P300 event, which are the oriented attention and working memory [58], are substantially preserved. More complex oddball paradigms, including the P300a detection, could improve the exploration of initial cognitive dysfunction in early MS [63].

Interestingly, we found that a higher level of fatigue scores corresponded to reduced P300 amplitude in pwMS and HCs. A recent work [29] tried to assess whether fatigue affected ERP findings in patients with RRMS, demonstrating a positive correlation between fatigue and reaction times.

Considering that fatigue already prevailed in pwMS in the early phase as compared to HCs, we can suppose that the progression of the disease could cause a decrement of cognitive efficiency in the course of task performance with consequent P300 amplitude reduction.

This was a pilot study whose results are surely affected by the small sample size, made even smaller by the exclusion of five controls and two patients because of a technical problem that occurred in the codification of the motor response. The high spatial resolution of scalp potentials and fields did not indicate a trend toward amplitude reduction in any time frame. However, further evaluation of P300 source generation on realistic MRI models would possibly display mechanisms of cortical rearrangement in the presence of initial cortical dysfunction.

Although the present results do not support P300 as a clear biomarker of cognitive impairment, normal amplitude and scalp distribution as displayed in hd MEG analysis could be related to early remodeling in cortical synapsis.

## 5. Conclusions

Characterizing subtle symptoms in MS is a complex task, although some neurophysiological hallmarks could be employed to assess information processing and provide more information on the involvement of higher brain functions. The present pilot study indicated that early pwMS, DMDs naive did not display an amplitude reduction and different scalp distribution of ERP and ERF related to an acoustic oddball paradigm. Phenomena of cortical adaptation to early dysfunction could preserve the cognitive performance of the P300 acoustic task, as well as related cortical responses. Further studies will be designed to clarify possible modification of source generators caused by cortical dysfunction. Based on the present data, we cannot suppose that P300 amplitude reduction is a hallmark of early cortical impairment in pwMS. Moreover, the development of fatigue could prospectively lead to ERP amplitude decline, applying it as an objective measure of this commonly reported symptom in MS.

## Figures and Tables

**Table 1:** Demographic and clinical variables of participant groups.

	Patient (*n* = 16)	Controls (*n* = 19)	*p* Value
Sex, *n*° (%)			
F	14 (87.50%)	13 (68.42%)	0.19 ^×^
M	2 (12.50%)	6 (31.58%)	
Age (years) *	36.5 ± 7.5	36.0 ± 16.0	0.98 °
Education (years) *	13 ± 1	13 ± 4	0.49 °
Duration of disease (months) *	11.44 ± 19.96		
EDSS *	2.0 ± 1		
Clinical phenotype			
CIS, *n*° (%)	1 (6.25%)		
RRMS, *n*° (%)	15 (93.75%)		

^×^ = Chi square value; ° = ANOVA F values; *n*°, number; *, median ± interquartile range; EDSS, Expanded Disability Status Scale; CIS, Clinically isolated syndrome; RRMS, relapsing-remitting multiple sclerosis.

**Table 2:** Mean values and statistical analysis of cognitive tests, fatigue severity scale inventory (FSS), and Beck Depression Inventory (BDI) between 16 pwMS and 19 HCs.

	Mean	Error DS	95% CI		ANOVA
			Lower	Upper	
PASAT					
Control	140.000	9.162	121.338	158.662	F 5.21
Patient	109.500	9.717	89.706	129.294	P 0.029
SDMT					
Control	52.611	3.303	45.884	59.339	F 1.32
Patient	47.063	3.503	39.927	54.198	P 0.25
FSS					
Control	1.139	0.408	0.308	1.970	F 6.29
Patient	2.631	0.433	1.750	3.513	P 0.017
BDI					
Control	2.72	1.13	0.4	5.04	F 4.51
Patient	6.25	1.21	3.79	8.71	P 0.041

PASAT, Paced Auditory Serial Addition Tests; SDMT, Symbol Digit Modality Test.

**Table 3:** Differences in global cognitive domain scores between Multiple Sclerosis patients (pwMS) and healthy controls (HCs).

	pw (*n* = 16)	HCs (*n* = 15)	*p*-Value
Attention and processing speed	121 [122–142]	157 [125.5–165]	*p* < 0.01
Memory	105 [84.5–110]	122 [101–135]	*p* = 0.16
Verbal Fluency	19 [15–20.5]	23 [19–25]	*p* = 0.08
Executive Functions	122 [75–131]	83 [66.5–84]	*p* = 0.44

**Table 4:** Mean values and standard deviation of P300 amplitude and latency measured on Pz electrode. Results of one-way ANOVA are reported.

	Patients (*n* = 16)	Controls (*n* = 19)	ANOVA: F	*p*-Value
P300 latency (msec)	326.15 ± 28.85	311.37 ± 31.3	1.66	0.21
P300 amplitude (uV)	5.7 ± 4	6.7 ± 3.4	0.61	0.4

## Data Availability

All clinical and neurophysiological data are available on request.

## Supplementary Materials

The following are available online at https://www.mdpi.com/article/10.3390/brainsci11040481/s1, Figure S1: Morlet wavelet. The complex Morlet wavelet is a Gaussian weighted sinusoid (blue for real values, red for imaginary values). It has a point spread function with Gaussian shape both in time (temporal resolution) and in frequency (spectral resolution). Resolution is given in units of FWHM (full width half maximum) for several frequencies. Figures S2–S3: Mean values and confidence intervals of latency (Figure S1) and amplitude (Figure S2) of the main ERPs by oddball paradigm in pwMS and HCs. Figure S4: Topographical representation of Grand Average of ERP-ERF peaks in 16 pwMS (above) and 19 HCs (below). From left to right: EEG-ERP, MEG-magnetometers-ERF, MEG-gradiometers-ERF. (a) N1 component; (b) P2 component; (c) N2 component. Figure S5: Deviant trial. Representative parietal midline gradiometer (a,b), magnetometer (c,d), and EEG derivation (e,f) for controls (top) and patients (bottom) The gradiometers showed a not significant increase in negative modulation induced by deviant stimulus in patients as compared to controls.

## References

[1] Chiaravalloti N.D., DeLuca J. (2008). Cognitive impairment in multiple sclerosis. Lancet Neurol..

[2] Brochet B., Ruet A. (2019). Cognitive Impairment in Multiple Sclerosis With Regards to Disease Duration and Clinical Phenotypes. Front. Neurol..

[3] Ruano L., Portaccio E., Goretti B., Niccolai C., Severo M., Patti F., Cilia S., Gallo P., Grossi P., Ghezzi A. (2017). Age and disability drive cognitive impairment in multiple sclerosis across disease subtypes. Mult. Scler..

[4] Ruano L., Portaccio E., Goretti B., Niccolai C., Severo M., Patti F., Cilia S., Gallo P., Grossi P., Ghezzi A. (2007). Early cognitive impairment in patients with clinically isolated syndrome suggestive of multiple sclerosis. Mult. Scler..

[5] Schulz D., Kopp B., Kunkel A., Faiss J.H. (2006). Cognition in the early stage of multiple sclerosis. J. Neurol..

[6] Rao S.M., Leo G.J., Bernardin L., Unverzagt F. (1991). Cognitive dysfunction in multiple sclerosis. Frequency patterns and prediction. Neurology.

[7] Zipoli V., Goretti B., Hakiki B., Siracusa G., Sorbi S., Portaccio E., Amato M.P. (2010). Cognitive impairment predicts conversion to multiple sclerosis in clinically isolated syndromes. Mult. Scler..

[8] Pitteri M., Romualdi C., Magliozzi R., Monaco S., Calabrese M. (2017). Cognitive impairment predicts disability progression and cortical thinning in MS: An 8-year study. Mult. Scler..

[9] Ghezzi A., Goretti B., Portaccio E., Roscio M., Amato M.P. (2010). Cognitive impairment in pediatric multiple sclerosis. Neurol. Sci..

[10] Rao S.M., Leo G.J., Ellington L., Nauertz T., Bernardin L., Unverzagt F. (1991). Cognitive dysfunction in multiple sclerosis. II. Impact on employment and social functioning. Neurology.

[11] Ruet A., Deloire M., Hamel D., Ouallet J.C., Petry K., Brochet B. (2013). Cognitive impairment, health-related quality of life and vocational status at early stages of multiple sclerosis: A 7-year longitudinal study. J. Neurol..

[12] Sumowski J.F., Benedict R., Enzinger C., Filippi M., Geurts J.J., Hamalainen P., Hulst H., Inglese M., Leavitt V.M., Rocca M.A. (2018). Cognition in multiple sclerosis: State of the field and priorities for the future. Neurology.

[13] Sumowski J.F., Benedict R., Enzinger C., Filippi M., Geurts J.J., Hamalainen P., Hulst H., Inglese M., Leavitt V.M., Rocca M.A. (2019). Pathophysiological and cognitive mechanisms of fatigue in multiple sclerosis. J. Neurol. Neurosurg. Psychiatry.

[14] Bisecco A., Caiazzo G., d’Ambrosio A., Sacco R., Bonavita S., Docimo R., Cirillo M., Pagani E., Filippi M., Esposito F. (2016). Fatigue in multiple sclerosis: The contribution of occult white matter damage. Mult. Scler..

[15] Gobbi C., Rocca M.A., Riccitelli G., Pagani E., Messina R., Preziosa P., Colombo B., Rodegher M., Falini A., Comi G. (2014). Influence of the topography of brain damage on depression and fatigue in patients with multiple sclerosis. Mult. Scler..

[16] Calabrese M., Rinaldi F., Grossi P., Mattisi I., Bernardi V., Favaretto A., Perini P., Gallo P. (2010). Basal ganglia and frontal/parietal cortical atrophy is associated with fatigue in relapsing-remitting multiple sclerosis. Mult. Scler..

[17] Cruz Gómez Á.J., Ventura Campos N., Belenguer A., Ávila C., Forn C. (2013). Regional brain atrophy and functional connectivity changes related to fatigue in multiple sclerosis. PLoS ONE.

[18] Pittion-Vouyovitch S., Debouverie M., Guillemin F., Vandenberghe N., Anxionnat R., Vespignani H. (2006). Fatigue in multiple sclerosis is related to disability, depression and quality of life. J. Neurol. Sci..

[19] Kos D., Kerckhofs E., Nagels G., D’hooghe M.B., Ilsbroukx S. (2008). Origin of fatigue in multiple sclerosis: Review of the literature. Neurorehabil. Neural Repair.

[20] Strober L.B., Arnett P.A. (2005). An examination of four models predicting fatigue in multiple sclerosis. Arch. Clin. Neuropychol..

[21] Biberacher V., Schmidt P., Selter R.C., Pernpeinter V., Kowarik M.C., Knier B., Buck D., Hoshi M.M., Korn T., Berthele A. (2018). Fatigue in multiple sclerosis: Associations with clinical, MRI and CSF parameters. Mult. Scler..

[22] Iancheva D., Trenova A., Mantarova S., Terziyski K. (2019). Functional Magnetic Resonance Imaging Correlations Between Fatigue and Cognitive Performance in Patients With Relapsing Remitting Multiple Sclerosis. Front. Psychiatry.

[23] Krupp L.B., LaRocca N.G., Muir-Nash J., Steinberg A.D. (1989). The fatigue severity scale. Application to patients with multiple sclerosis and systemic lupus erythematosus. Arch. Neurol..

[24] Penner I.K., Raselli C., Stöcklin M., Opwis K., Kappos L., Calabrese P. (2009). The Fatigue Scale for Motor and Cognitive Functions (FSMC): Validation of a new instrument to assess multiple sclerosis-related fatigue. Mult. Scler..

[25] Gross J. (2019). Magnetoencephalography in Cognitive Neuroscience: A Primer. Neuron.

[26] Tewarie P., Schoonheim M.M., Schouten D.I., Polman C.H., Balk L.J., Uitdehaag B.M., Geurts J.J., Hillebrand A., Barkhof F., Stam C.J. (2015). Functional brain networks: Linking thalamic atrophy to clinical disability in multiple sclerosis, a multimodal fMRI and MEG study. Hum. Brain Mapp..

[27] Pokryszko-Dragan A., Zagrajek M., Slotwinski K., Bilinska M., Gruszka E., Podemski R. (2016). Event-related potentials and cognitive performance in multiple sclerosis patients with fatigue. Neurol. Sci..

[28] Chinnadurai S.A., Venkatesan S.A., Shankar G., Samivel B., Ranganathan L.N. (2016). A study of cognitive fatigue in multiple sclerosis with novel clinical and electrophysiological parameters utilizing the event related potential P300. Mult. Scler. Relat. Disord..

[29] Lazarevic S., Azanjac Arsic A., Aleksic D., Toncev G., Miletic-Drakulic S. (2021). Depression and Fatigue in Patients with Multiple Sclerosis Have No Influence on the Parameters of Cognitive Evoked Potentials. J. Clin. Neurophysiol..

[30] Aminoff J.C., Goodin D.S. (2001). Long-latency cerebral event-related potentials in multiple sclerosis. J. Clin. Neurophysiol..

[31] Gil R., Zai L., Neau J.P., Jonveaux T., Agbo C., Rosolacci T., Burbaud P., Ingrand P. (1993). Event-related auditory evoked potentials and multiple sclerosis. Electroencephalogr. Clin. Neurophysiol..

[32] Piras M.R., Magnano I., Canu E.D., Paulus K.S., Satta W.M., Soddu A., Conti M., Achene A., Solinas G., Aiello I. (2003). Longitudinal study of cognitive dysfunction in multiple sclerosis: Neuropsychological, neuroradiological, and neurophysiological findings. J. Neurol. Neurosurg. Psychiatry.

[33] Ivica N., Titlic M., Pavelin S. (2013). P300 wave changes in patients with multiple sclerosis. Acta Inform. Med..

[34] Giesser B.S., Schroeder M.M., LaRocca N.G., Kurtzberg D., Ritter W., Vaughan H.G., Scheinberg L.C. (1992). Endogenous event-related potentials as indices of dementia in multiple sclerosis patients. Electroencephalogr. Clin. Neurophysiol..

[35] Ellger T., Bethke F., Frese A., Luettmann R.J., Buchheister A., Ringelstein E.B., Evers S. (2002). Event-related potentials in different subtypes of multiple sclerosis—A cross-sectional study. J. Neurol. Sci..

[36] Triantafyllou N.I., Voumvourakis K., Zalonis I., Sfagos K., Mantouvalos V., Malliara S., Papageorgiou C. (1992). Cognition in relapsing-remitting multiple sclerosis: A multichannel event-related potential (P300) study. Acta Neurol. Scand..

[37] Honig L.S., Ramsay R.E., Sheremata W.A. (1992). Event-related potential P300 in multiple sclerosis. Relation to magnetic resonance imaging and cognitive impairment. Arch. Neurol..

[38] Magnano I., Aiello I., Piras M.R. (2006). Cognitive impairment and neurophysiological correlates in MS. J. Neurol. Sci..

[39] Zwecker M., Sarova I., Lavie M., Zeilig G., Achiron A. (2018). Detection of Cognitive Impairment in Multiple Sclerosis Based on P300 Event-Related Potential. Int. J. Phys. Med. Rehabil..

[40] Magnié M.N., Bensa C., Laloux L., Bertogliati C., Faure S., Lebrun C. (2007). Contribution of cognitive evoked potentials for detecting early cognitive disorders in multiple sclerosis. Rev. Neurol..

[41] Kocer B., Unal T., Nazliel B., Biyikli Z., Yesilbudak Z., Karakas S., Irkec C. (2008). Evaluating sub-clinical cognitive dysfunction and event-related potentials (P300) in clinically isolated syndrome. Neurol. Sci..

[42] Pokryszko-Dragan A., Dziadkowiak E., Zagrajek M., Slotwinski K., Gruszka E., Bilinska M., Podemski R. (2016). Cognitive performance, fatigue and event-related potentials in patients with clinically isolated syndrome. Clin. Neurol. Neurosurg..

[43] Kiiski H., Reilly R.B., Lonergan R., Kelly S., O’Brien M.C., Kinsella K., Bramham J., Burke T., ODonnchadha S., Nolan H. (2012). Only low frequency event-related EEG activity is compromised in multiple sclerosis: Insights from an independent component clustering analysis. PLoS ONE.

[44] Sundgren M., Nikulin V.V., Maurex L., Wahlin L., Piehl F., Brismar T. (2015). P300 amplitude and response speed relate to preserved cognitive function in relapsing-remitting multiple sclerosis. Clin. Neurophysiol..

[45] Whelan R., Lonergan R., Kiiski H., Nolan H., Kinsella K., Bramham J., O’Brien M., Reilly R.B., Hutchinson M., Tubridy N. (2010). A high-density ERP study reveals latency, amplitude and topo graphical differences in multiple sclerosis patients versus controls. Clin. Neurophysiol..

[46] Kurtzke J.F. (1983). Rating neurologic impairment in multiple sclerosis: An expanded disability status scale (EDSS). Neurology.

[47] Sanders E.A., Reulen J.P., Van der Velde E.A., Hogenhuis L.A. (1986). The diagnosis of multiple sclerosis. Contribution of non clinical tests. J. Neurol. Sci..

[48] Hase Y., Horsburgh K., Ihara M., Kalaria R.N. (2018). White matter degeneration in vascular and other ageing-related dementias. J. Neurochem..

[49] Lublin F.D. (2014). New multiple sclerosis phenotypic classification. Eur. Neurol..

[50] Thompson A.J., Banwell B.L., Barkhof F., Carroll W.M., Coetzee T., Comi G., Correale J., Fazekas F., Filippi M., Freedman M.S. (2018). Diagnosis of multiple sclerosis: 2017 revisions of the McDonald criteria. Lancet Neurol..

[51] Olsson T., Barcellos L.F., Alfredsson L. (2017). Interactions between genetic, lifestyle and environmental risk factors for multiple sclerosis. Nat. Rev. Neurol..

[52] Boringa J.B., Lazeron R.H., Reuling I.E., Adèr H.J., Pfennings L., Lindeboom J., de Sonneville L.M., Kalkers N.F., Polman C.H. (2001). The brief repeatable battery of neuropsychological tests: Normative values allow application in multiple sclerosis clinical practice. Mult. Scler..

[53] Tombaugh T.N. (2004). Trail Making Test A and B: Normative data stratified by age and education. Arch. Clin. Neuropsychol..

[54] Amato M.P., Portaccio E., Goretti B., Zipoli V., Ricchiuti L., De Caro M.F., Patti F., Vecchio R., Sorbi S., Trojano M. (2006). The Rao’s Brief Repeatable Battery and Stroop Test: Normative values with age, education and gender corrections in an Italian population. Mult. Scler..

[55] Beck A.T., Steer R.A., Brown G.K. (1996). Manual for Beck Depression Inventory-II.

[56] Maris E., Oostenveld R. (2007). Nonparametric statistical testing of EEG- and MEG-data. J. Neurosci. Methods.

[57] Polman C.H., Reingold S.C., Banwell B., Clanet M., Cohen J.A., Filippi M., Fujihara K., Havrdova E., Hutchinson M., Kappos L. (2011). Diagnostic criteria for multiple sclerosis: 2010 revisions to the McDonald criteria. Ann. Neurol..

[58] Waliszewska-Prosół M., Nowakowska-Kotas M., Kotas R., Bańkowski T., Pokryszko-Dragan A., Podemski R. (2018). The relationship between event-related potentials, stress perception and personality type in patients with multiple sclerosis without cognitive impairment: A pilot study. Adv. Clin. Exp. Med..

[59] Artemiadis A.K., Anagnostouli M.C., Zalonis I.G., Chairopoulos K.G., Triantafyllou N.I. (2018). Structural MRI Correlates of Cognitive Event-Related Potentials in Multiple Sclerosis. J. Clin. Neurophysiol..

[60] de Tommaso M., Betti V., Bocci T., Bolognini N., Di Russo F., Fattapposta F., Ferri R., Invitto S., Koch G., Miniussi C. (2020). Pearls and pitfalls in brain functional analysis by event-related potentials: A narrative review by the Italian Psychophysiology and Cognitive Neuroscience Society on methodological limits and clinical reliability-part I. Neurol. Sci..

[61] de Tommaso M., Betti V., Bocci T., Bolognini N., Di Russo F., Fattapposta F., Ferri R., Invitto S., Koch G., Miniussi C. (2020). Pearl and pitfalls in brain functional analysis by event-related potentials: A narrative review by the Italian Psychophysiology and Cognitive Neuroscience Society on methodological limits and clinical reliability-part II. Neurol. Sci..

[62] Linden D.E.J. (2005). The P300: Where in the Brain Is It Produced and What Does It Tell Us?. Neuroscientist.

[63] Kiiski H., Whelan R., Lonergan R., Nolan H., Kinsella K., Hutchinson M., Tubridy N., Reilly R.B. (2011). Preliminary evidence for correlation between PASAT performance and P3a and P3b amplitudes in progressive multiple sclerosis. Eur. J. Neurol..

