# Supplementary material for: Magnetoencephalography and High-Density Electroencephalography Study of Acoustic Event Related Potentials in Early Stage of Multiple Sclerosis: A Pilot Study on Cognitive Impairment and Fatigue
Source: Brain Sci. 2021 Apr 9;11(4):481. doi: 10.3390/brainsci11040481 (PMC8069556; doi:10.3390/brainsci11040481)
Supplement: Supplementary file 1 [file brainsci-11-00481-s001.pdf]

Figure S1-S2: Mean values and confidence intervals of latency (Figure S1) and amplitude (Figure S2) of main ERPs by odd ball paradigm in pwMS and HCs.

Figure S1

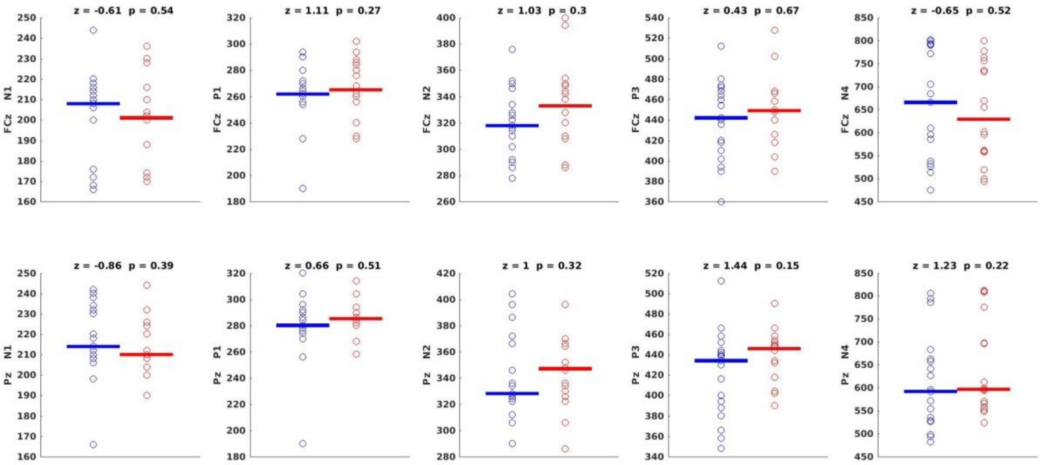

Figure S2

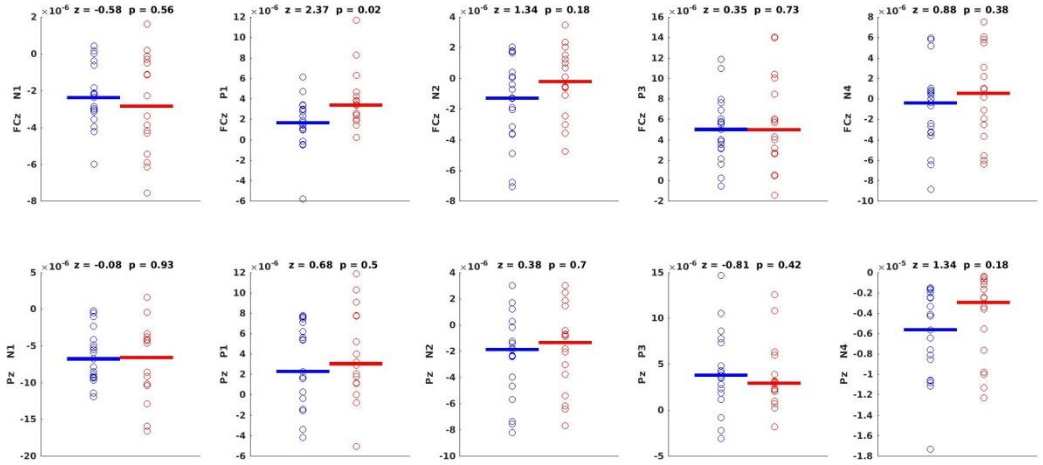

Figure S3. Topographical representation of Grand Average of ERP-ERF peaks in 16 pwMS (above) and 19 HCs (below).

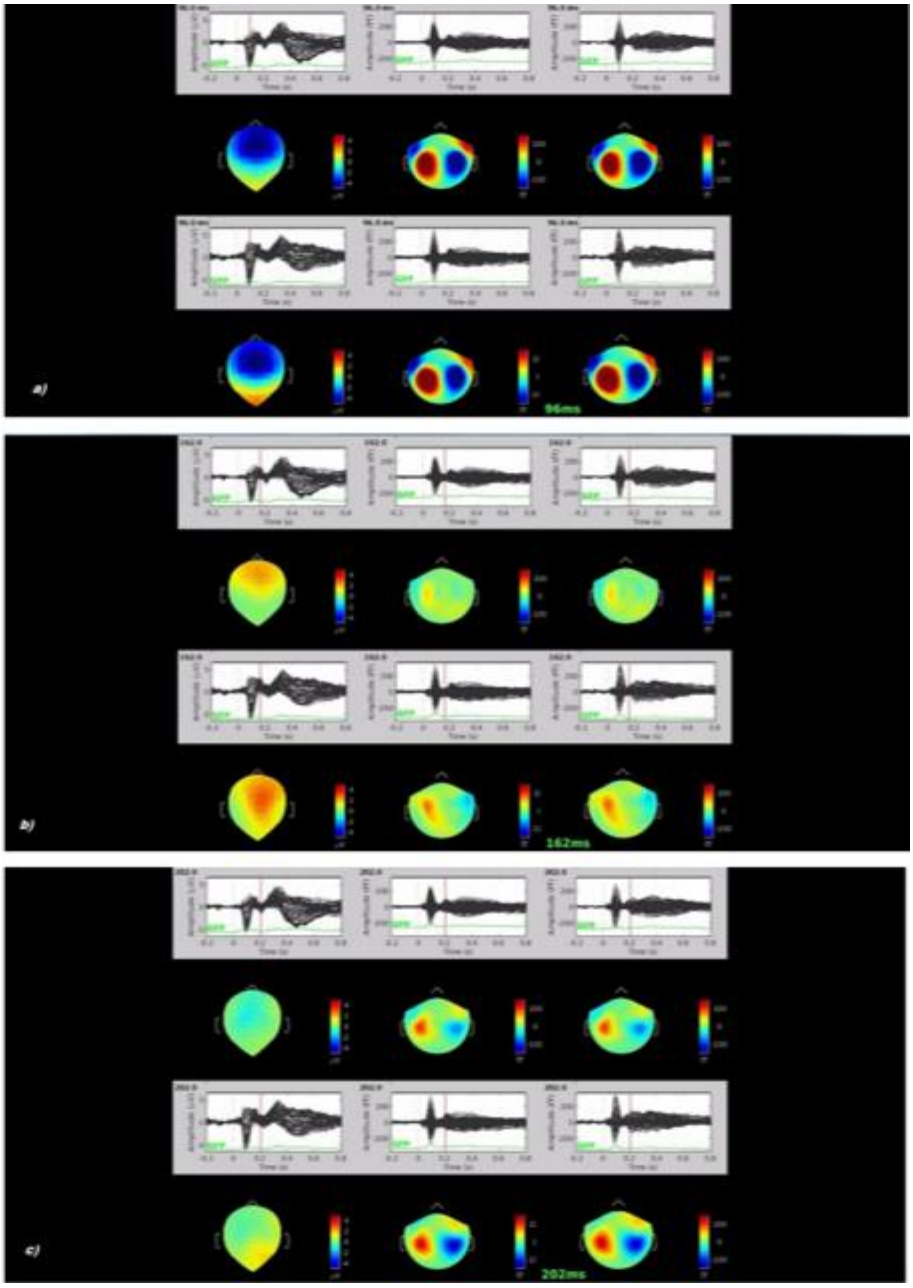

Fom left to right: EEG-ERP, MEG-magnetometers-ERF, MEG-gradiometers-ERF.a) N1 component; b) P2 component; c) N2 component

Figure S4 Deviant trial.

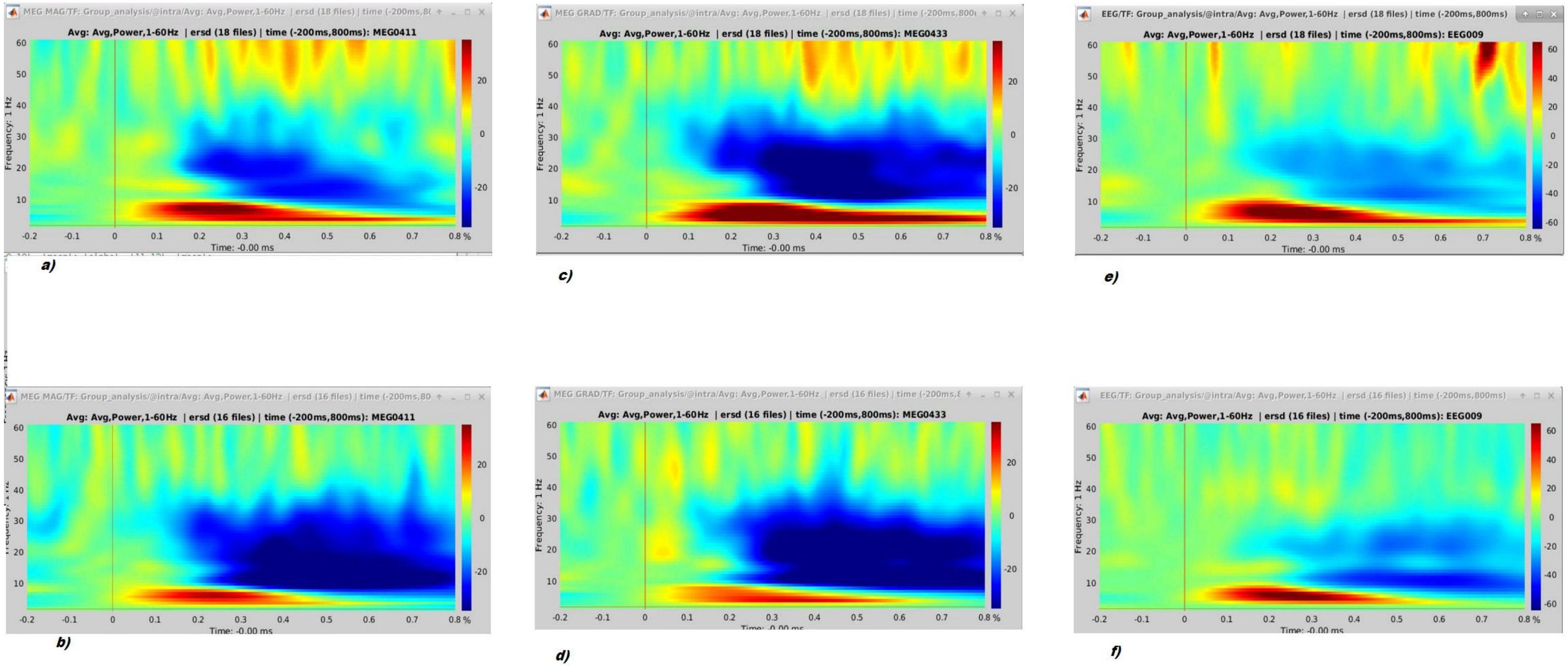

Representative parietal midline gradiometer (a,b), magnetometer (c,d) and EEG derivation (e,f) for controls (top) and patients (bottom) The gradiometers showed a not significant increase of negative modulation induced by deviant stimulus in patients as compared to controls.
